# Correlation between total phenolic and flavonoid contents and biological activities of 12 ethanolic extracts of Iranian propolis

**DOI:** 10.1002/fsn3.3356

**Published:** 2023-04-21

**Authors:** Shahnaz Fathi Hafshejani, Safa Lotfi, Elham Rezvannejad, Mojtaba Mortazavi, Ali Riahi‐Madvar

**Affiliations:** ^1^ Department of Biotechnology, Institute of Science and High Technology and Environmental Sciences Graduate University of Advanced Technology Kerman Iran; ^2^ Department of Molecular and Cell Biology, Faculty of Basic Sciences Kosar University of Bojnord Bojnord Iran

**Keywords:** anticholinesterase activity, molecular docking, propolis, total flavonoid content, total phenolic content

## Abstract

Propolis is a resinous substance produced by honey bees that is very popular as a natural remedy in traditional medicine. The current research is the first study on the biological properties of ethanolic extracts of propolis (EEP) from several different regions (12) of Iran. Total phenolic and flavonoid contents (TPC and TFC) of Iranian EEPs were variable between 26.59–221.38 mg GAE/g EEP and 4.8–100.03 mg QE/g EEP. The DPPH scavenging assay showed all the studied EEP samples, except for the sample with the lowest TPC and TFC (P6), have suitable antioxidant activity. All the EEPs inhibited both cholinesterase enzymes (acetylcholinesterase: AChE, butyrylcholinesterase: BuChE) but most of them exhibited a distinct selectivity over BuChE. Evaluation of the antibacterial activity of the EEP samples using four pathogenic bacteria (*B. cereus*, *S. aureus*, *A. baumannii*, and *P. aeruginosa*) demonstrated that the antibacterial properties of propolis are more effective on the gram‐positive bacterium. Spearman correlation analysis showed a strong positive correlation between TPC and TFC of the Iranian EEPs and their antioxidant, anticholinesterase, and antibacterial activities. Considering that there is ample evidence of anticholinesterase activity of flavonoids and a significant correlation between the anticholinesterase activity of the studied Iranian EEPs and their total flavonoid content was observed, the interaction of 17 well‐known propolis flavonoids with AChE and BuChE was explored using molecular docking. The results indicated that all the flavonoids interact with the active site gorge of both enzymes with high affinity. Summing up, the obtained results suggest that Iranian propolis possesses great potential for further studies.

## Graphical abstract

The current research is the first study on the biological properties of ethanolic extracts of propolis (EEP) from several different regions (12) of Iran. According to the obtained data, there is a strong positive correlation between TPC and TFC of the Iranian EEPs and their antioxidant, anticholinesterase, and antibacterial activities.

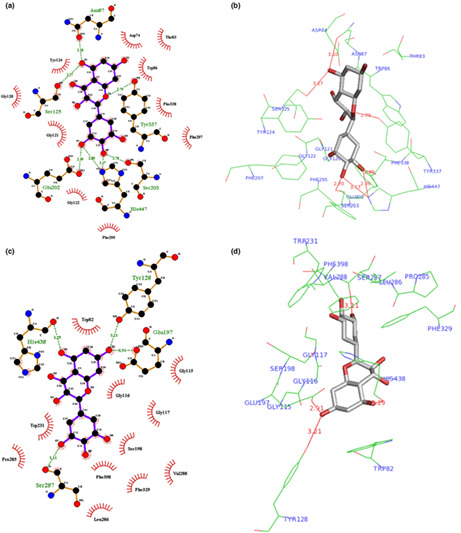

## 1 INTRODUCTION

Propolis (bee glue) is a natural product created by honey bees (*Apis mellifera* L.) by mixing their saliva, beeswax, and exudates derived from different parts of the plants (Marcucci, 1995; Silva‐Carvalho et al., 2015). This resinous material forms the inner lining of the beehives and is used as a protective barrier against foreign invaders, wind, and rain. The honey bees also use propolis to repair cracks and holes in the walls of the hive and seal openings and smooth the inner walls, maintain the hive's internal temperature and cover (embalm) the corpses of large invaders that are difficult to transport out of the hive. Propolis has a very pleasant aroma and due to its antimicrobial activity, prevents the growth of bacteria and fungi inside the hives (Burdock, 1998; Martinotti & Ranzato, 2015).

The exact composition, aroma, and color of raw propolis (green, red, brown, and yellow) depend on several factors such as botanical source, collection season, and geographical area (Wang et al., 2016). Propolis is mainly composed of resin (50%), wax (30%), essential oils (10%), pollen (5%), and other organic compounds (5%) (Gómez‐Caravaca et al., 2006).

Over 180 compounds, mostly polyphenols including flavonoids, phenolic acids, esters, phenolic aldehydes, and ketones have been identified as components of propolis samples originating from different geographical regions of the world. Polyphenol content is often considered an indicator to assess the quality of propolis. Two phenolic acids (caffeic acid and cinnamic acid), 12 different flavonoids (chrysin, rutin, catechin, galangin, myricetin, luteolin, quercetin, naringenin, pinocembrin, acacetin, kaempferol, and apigenin), and one stilbene derivative (resveratrol) were identified in propolis extracts using capillary zone electrophoresis. Terpenes are responsible for the resinous odor of propolis and sometimes are used as a criterion for distinguishing between premium and ordinary propolis (Bhargava et al., 2021; Volpi, 2004). α‐amyrin, β‐amyrin, isocupressic acid, geraniol, limonene, and lupeol are among the terpene compounds identified in propolis (Šturm & Poklar Ulrih, 2020). Propolis also possesses biometabolites (sugars, amino acids, lipids, and nucleic acids), hydrocarbons (monoesters, diesters, aromatic esters, alkanes, alkenes, and alkadienes), important vitamins (B1, B2, B6, C, and E), and useful minerals (calcium, potassium, magnesium, sodium, iron, copper, zinc, and manganese). There are also a small number of enzymes in propolis including glucose 6‐phosphatase, succinic dehydrogenase, acid phosphatase, adenosine triphosphatase, and beta‐amylase (Bhargava et al., 2021; Lotfy, 2006).

Propolis has been used by humankind for different medicinal and nonmedicinal purposes since ancient times (Kuropatnicki et al., 2013). According to the published scientific data, various biological properties have been attributed to propolis, such as anticancer, antioxidant, anticholinesterase, antihypertensive, liver protection, wound‐healing, oral health, anti‐inflammatory, antiulcer, immunomodulatory, antimicrobial, antiviral, antifungal, and antiparasitic (Baltas et al., 2016; Bhargava et al., 2021; Dilokthornsakul et al., 2022; Kocot et al., 2018; Pasupuleti et al., 2017; Rezvannejad et al., 2017; Saeed et al., 2021; Suran et al., 2021; Viuda‐Martos et al., 2008). The data provided by in vitro studies, animal models, and human clinical trials demonstrate that propolis can reduce the manifestations of neurological and brain disorders through its protective and therapeutic effects (Zulhendri, Chandrasekaran, et al., 2021; Zulhendri, Perera, et al., 2021).

The current research is the first comprehensive study on the biological properties of several propolis samples collected from different regions of Iran. This study covers the evaluation of the total phenolic and flavonoid contents (TPC and TFC) and the antioxidant, anticholinesterase, and antibacterial activities of 12 ethanolic extracts of Iranian propolis. Although the antibacterial and antioxidant activities of Iranian propolis have been previously studied, its anticholinesterase activity has not been studied until now. Meanwhile, previous studies have been conducted on one or a few limited Iranian propolis samples. In this research, also for the first time, the interaction of 17 well‐known propolis flavonoids with the cholinesterase enzymes (AChE and BuChE) has been investigated using molecular docking.

## 2 MATERIALS AND METHODS

### 2.1 Chemicals

Electric eel acetylcholinesterase (Type VI‐S), acetylthiocholine iodide, equine serum butyrylcholinesterase, *S*‐butyrylthiocholine iodide, 5,5′‐dithiobis‐(2‐nitrobenzoic acid) (DTNB), neostigmine, 2, 2‐diphenyl‐1‐picrylhydrazyl (DPPH), Folin–Ciocalteu reagent, gallic acid, quercetin, ascorbic acid, ciprofloxacin, Luria‐Bertani (LB) and nutrient agar media were purchased from Sigma‐Aldrich. Ethanol, Na_2_CO_3_, KH_2_PO_4_, K_2_HPO_4_, aluminum chloride, and methanol were provided by Merck.

### 2.2 Propolis samples and preparation of the ethanolic extracts

Twelve Iranian propolis samples (P1–P12) used in this research are listed in Table 1, along with coordinates. Raw propolis samples were collected by experienced beekeepers from beehives located in various regions of Iran. The propolis samples were stored at −20°C until use. Preparation of the ethanolic extracts of propolis (EEP) was performed in the following steps: Raw propolis was pulverized by liquid nitrogen. Ten grams of powdered propolis with 100 mL of 80% ethanol was placed in a dark glass flask and stirred on a shaker at room temperature for 72 h and the mixture was then filtrated by centrifugation in two steps. The supernatant was concentrated using rotary evaporation at 40°C and then placed in a vacuum oven at 40°C for complete drying. The ethanolic extracts were kept in the dark containers at −20°C until further steps.

**TABLE 1:** The Propolis samples studied in this research were collected from 12 various geographical regions of Iran.

Propolis sample	Geographical region	Coordinates
P1	Ardebil	38.2537° N, 48.3000° E
P2	Shahrekord	32.3282° N, 50.8769° E
P3	Najafabad	32.6382° N, 51.3575° E
P4	Lalehzar	29.513894° N, 56.82268° E
P5	Rayen	29.5913° N, 57.4458° E
P6	Lordegan	31.5166° N, 50.8144° E
P7	Qom	34.6416° N, 50.8746° E
P8	Gorgan	36.8418° N, 54.4334° E
P9	Binalud	36.4264° N, 58.8492° E
P10	Daregaz	37.4455° N, 59.1099° E
P11	Khoy	38.5534° N, 44.9397° E
P12	Kerman	30.2793° N, 57.1365° E

### 2.3 Determination of total phenolic content

The total phenolic content (TPC) of the EEP samples was measured utilizing the colorimetric Folin–Ciocalteu method and gallic acid was used as the calibration standard (Singleton & Rossi, 1965). The results were expressed as milligrams of gallic acid equivalents (GAE) per gram of EEP (mg GAE/g EEP). For each sample, the experiment was performed in triplicate. For this purpose, 100 μL of 0.2 N Folin–Ciocalteu reagent, 590 μL of distilled water, and 10 μL of EEP in 80% ethanol (final concentration of 50 μg/mL) were placed in a cell for 1 min at room temperature in the dark. Three hundred microliters of sodium carbonate (7.5%) was then added to the cell and the mixture was incubated in the dark for 30 min at room temperature. The absorbance was read at 735 nm with a spectrophotometer (Cary 50, Australia). A solution containing 100 μL of 0.2 N Folin–Ciocalteu reagent, 600 μL of distilled water, and 300 μL of sodium carbonate (7.5%) was utilized as the blank sample.

### 2.4 Determination of total flavonoid content

The aluminum chloride colorimetric method was used to measure the total flavonoid content (TFC) of EEP samples (Wang et al., 2016). Five hundred microliters of EEP in 75% ethanol (final concentration of 50 μg/mL) was added to 500 μL of 2% aluminum chloride in a tube. The tube was then incubated in the dark for 15 min at room temperature. The absorbance was recorded at 435 nm with a spectrophotometer (Cary 50, Australia). A solution containing 500 μL of 2% aluminum chloride and 500 μL of distilled water was used as the blank. Three replications were performed for each sample. Quercetin was applied as the calibration standard and the TFC of EEP samples was expressed in milligrams of quercetin equivalents (QE) per gram of EEP (mg QE/g EEP).

### 2.5 DPPH‐free radical scavenging assay

To evaluate the antioxidant activity of EEP samples, the DPPH (2, 2‐diphenyl‐1‐picrylhydrazyl) free radical scavenging assay was used (Bondet et al., 1997). All the EEP samples were tested at six different concentrations. For each concentration, three replications were performed. Briefly, 750 μL of 0.4 mM DPPH solution was added to 250 μL of EEP dissolved in methanol. After 30 min of incubation in the dark at room temperature, the absorbance of the sample was recorded at 517 nm. A sample containing 750 μL of DPPH solution (0.4 mM) and 250 μL of methanol was considered as the control sample. Ascorbic acid was used as the positive control. For this aim, six different concentrations of ascorbic acid (0.5–12.5 μg/mL) were used and for each concentration, three replications were considered.

The following formula was used to calculate the percentage of DPPH inhibition free radical by each concentration of the EEP sample:

$$\text{Inhibition of DPPH}\;\left(\%\right)=\left[\left(A_{\text{control}}-A_{\text{sample}}\right)/A_{\mathrm{control}}\right]\;\times \;100$$

*A*_control_, absorbance of the control sample; *A*_sample_, absorbance of the EEP sample.

The IC_50_ value (the concentration of a sample that has the ability to inhibit DPPH radical by 50%) for each sample was obtained using a dose–response graph plotting the percentage of DPPH inhibition versus the concentration logarithm of the EEP sample.

### 2.6 Measurement of anticholinesterase activity

To determine the half maximal inhibitory concentration (IC_50_) of EEP samples for acetylcholinesterase (AChE) or butyrylcholinesterase (BuChE), the activity of the enzymes was measured in the absence and presence of six different concentrations of each EEP sample by the Ellman method (Ellman et al., 1961). Each concentration was analyzed in triplicate and a well‐known inhibitor of the cholinesterase enzymes, neostigmine, was used as the positive control. All the enzyme assays were performed on a 96‐well plate with a final volume of 200 μL.

The assay mixture consisted of 0.1 M potassium phosphate buffer (pH 8.0) and the enzyme (AChE or BuChE) and DTNB with the final concentrations of 0.1 unit/mL and 0.5 mM, respectively. The EEPs (in 70% ethanol) were incubated with the assay mixture for 10 min. Substrate (acetylthiocholine iodide or S‐butyrylthiocholine iodide) was then added to the assay with the final concentration of 1 mM and the absorbance was read after 10 min at 405 nm by a microplate reader (Elx808 Biotek Instruments). A sample containing all assay components, except the enzyme was applied as the blank. Finally, the percentage of enzyme activity inhibition at each concentration of the EEP sample was determined and the IC_50_ value was calculated from the dose–response curve plotting the percentage of inhibition versus concentration logarithm of the EEP sample. The results were reported as the mean ± standard deviation (SD).

### 2.7 Evaluation of antibacterial activity

To evaluate the antibacterial activity of EEP samples, four pathogenic bacteria, *Bacillus cereus*, *Staphylococcus aureus*, *Acinetobacter baumannii*, *and Pseudomonas aeruginosa* were used. The first two bacteria are gram‐positive and the next two are gram‐negative. In the first step, the lowest inhibitory concentration (MIC: minimum inhibitory concentration) and the lowest bactericidal concentration (MBC: minimum bactericidal concentration) of each EEP sample were determined using the broth macrodilution method (Balouiri et al., 2016; Shohayeb et al., 2014). In brief, seven different concentrations from each EEP sample (in 70% ethanol) were prepared using the LB culture medium by two‐fold dilution in test tubes. A bacterial suspension equivalent to a 0.5 McFarland standard was added to each tube. The sample tubes, the positive control tube (bacterial suspension with LB culture medium), the negative control tube (LB culture medium and propolis extract), and the tube containing only LB culture medium were then incubated for 24 h at 37°C. After the incubation period, the results were investigated based on microbial turbidity. The lowest concentration in which microbial turbidity was not observed was considered MIC. Each test was repeated three times. In order to determine the lowest bactericidal concentration (MBC), the samples without microbial turbidity from the previous step (determination of MIC) were cultured in the solid LB medium, and the lowest concentration in which no bacterial colony was observed was considered MBC. Three replications were considered for each experiment.

In the next step, the antibacterial activity of the EPP samples was investigated using the agar well diffusion method (Balouiri et al., 2016; Domingue et al., 1994). For this purpose, seven different concentrations (similar to MIC determination) of each EEP sample were prepared using 70% ethanol by two‐fold dilution. The assay was performed in Petri dishes containing nutrient agar medium. After uniformly spreading the human pathogenic bacteria (*B. cereus*, *S. aureus*, *A. baumannii*, or *P. aeruginosa*) on the surface of the medium using a sterile swab, a well with a diameter of 5 mm was made in the medium and filled with 25 μL of the sample. The Petri dishes were then incubated at 37°C for 16 to 18 h. 70% ethanol solution and ciprofloxacin were used as the negative control and the positive control, respectively. For each EEP sample, three replicates per concentration were considered. The Diameter of the growth inhibition halo was measured for different concentrations of the EEP samples and 70% ethanol by caliper. To report the results, the diameter of the growth inhibition halo of each concentration of the EEP samples was subtracted from the halo diameter of 70% ethanol.

### 2.8 Statistical analysis

The data were calculated in the form of arithmetical mean values and standard deviations. The correlation between data was statistically analyzed using the one‐way ANOVA method, Spearman correlation of SPSS software (version 13.0). The diameter of growth inhibition halo data was also statistically analyzed using the ANOVA method and SAS software (version 9.1) and means comparison has been performed by Tukey method and proc GLM.

$$\text{Equation model}:\;y_{i}\;=\;\beta x_{i}+\;\varepsilon_{i}\;{}_{}$$

*y*_*i*_ denotes observations of studied parameters. *x*_*i*_ denotes different concentrations of the EEP samples. *ɛ*_*i*_ is the deviation vector.

### 2.9 Molecular docking studies

To explore the possible binding sites of 17 well‐known propolis flavonoids on the cholinesterase enzymes (AChE and BuChE) by molecular docking, AutoDock Vina 1.1.2 software (Trott & Olson, 2010) was used. For this aim, the crystal structure of human AChE (PDB entry 4M0E) (Cheung et al., 2013) and human BuChE (PDB entry 4TPK) (Brus et al., 2014) were used as the receptors. Before using the protein structures in the docking studies, the ligand and water molecules were removed and the missing residues were then added using MODELLER software (Webb & Sali, 2016). The protein structures were finally energy minimized by GROMACS (Berendsen et al., 1995). The SDF files of all 17 flavonoid compounds were downloaded from the PubChem website (https://pubchem.ncbi.nlm.nih.gov/) and then converted into PDB files using Mercury 1.4.2 software and ultimately energy minimized by ChemBio3D Ultra 14.0 software. AutoDock Tools 1.5.6 software was used to prepare the PDBQT files of the ligands and receptors. The position and dimensions of the grid boxes were selected in such a way that all parts of the active site gorge of the enzymes (catalytic triad, oxyanion hole, choline and acyl binding pockets, and PAS allosteric site) were included. The overall docking results were studied using AutoDock Tools. In order to analyze the results more accurately, the ligand‐receptor complex corresponding to the best docking conformation (lowest binding energy) was prepared for each flavonoid compound and analyzed using PyMol and LigPlot (Laskowski & Swindells, 2011) softwares and the amino acid residues involved in the binding process were determined.

## 3 RESULTS AND DISCUSSION

In this research, TPC and TFC and some biological activities (antioxidant, antimicrobial, and anticholinesterase) of 12 propolis samples collected from various geographical regions of Iran were investigated. The antibacterial and antioxidant properties of one or a few limited Iranian propolis samples have been previously studied (Afrouzan et al., 2018; Jafarzadeh Kashi et al., 2011; Mohammadzadeh et al., 2007; Rezvani et al., 2018; Samieerad & Gheibi, 2020). This research is therefore the first survey on the evaluation of the biological properties of several Iranian propolis samples and the first study on the anticholinesterase (anti‐AChE and anti‐BuChE) activity of Iranian propolis. In this study, also for the first time, the interaction of 17 well‐known flavonoid compounds of propolis with the active site gorge of the cholinesterase enzymes (AChE and BuChE) has been explored using molecular docking.

### 3.1 TPC, TFC, and antioxidant potency

Propolis consists of a wide array of polyphenolic compounds, mostly flavonoids, phenolic acids, and their esters. The flavonoid content is used as an index to evaluate the quality of propolis from temperate regions. Many biological and therapeutical properties of propolis are attributed to its phenolic and flavonoid composition (Huang et al., 2014; Wang et al., 2016). For example, the anticancer properties of propolis such as induction of apoptosis, anti‐angiogenic activity, and inhibition of proliferation, migration, and invasion are related to the polyphenolic compounds (Forma & Brys, 2021). According to the published papers, propolis polyphenolic compounds show anti‐inflammatory properties and can inhibit the synthesis of leukotrienes and prostaglandins and the activity of myeloperoxidase, ornithine decarboxylase, NADPH‐oxidase, and tyrosine‐protein kinase. These compounds also play essential roles in antioxidant, antibacterial, immunomodulatory, and wound‐healing activities (Oryan et al., 2018). Based on the literature, polyphenolic compounds of propolis possess cariogenic properties and reduce the accumulation of dental plaques and improve oral health (Kurek‐Gorecka et al., 2022).

The TPC and TFC of the studied Iranian EEP samples are shown in Table 2. As well seen, the TPC of the extracts are variable between 221.38 and 26.59 mg GAE/g EEP which is related to P9 and P6 samples, respectively. Among the 12 EEP samples, P9 and P6 also possess the highest (100.03 mg QE/g EEP) and lowest (4.8 mg QE/g EEP) TFC. A closer look at the results demonstrated that in addition to P9, the EEP samples of P8, P11, P4, P1, and P2 contain high TPC and TFC. As shown in Table 6, there is a perfect positive correlation between TPC and TFC (*R* ^2^ = 1), which means the EEP samples with higher TPCalso possess higher TFC and vice versa. There are frequent scientific reports that the vegetation of the propolis collection area has a very significant effect on its composition (Anjum et al., 2019; Huang et al., 2014; Wieczorek et al., 2022).

**TABLE 2:** Total phenolic and flavonoid contents (TPC and TFC) and DPPH IC_50_ values of 12 Iranian EEP samples (P1–P12). Ascorbic acid was used as a positive control in DPPH test.

Sample	TPC (mg GAE/g EEP)	TFC (mg QE/g EEP)	DPPH (IC_50_: μg/mL)
P1	148.33 ± 0.020	51.11 ± 0.004	29.50 ± 0.05
P2	140.78 ± 0.012	39.20 ± 0.016	32 ± 0.024
P3	52.61 ± 0.037	23.33 ± 0.016	64.49 ± 0.020
P4	165.96 ± 0.012	72.26 ± 0.004	5.64 ± 0.004
P5	70.25 ± 0.009	29.92 ± 0.021	44.47 ± 0.028
P6	26.59 ± 0.024	4.80 ± 0.008	1031.57 ± 1.25
P7	55.13 ± 0.021	27.30 ± 0.024	58 ± 0.046
P8	193.67 ± 0.028	84.16 ± 0.024	5.02 ± 0.004
P9	221.38 ± 0.008	100.03 ± 0.016	4.62 ± 0.008
P10	40.86 ± 0.020	21.98 ± 0.012	65.93 ± 0.038
P11	191.15 ± 0.024	76.23 ± 0.012	5.90 ± 0.004
P12	75.28 ± 0.046	33.88 ± 0.012	39.97 ± 0.024
Ascorbic acid	–	–	3.89 ± 0.028

Oxidative stress is mainly created as a result of the imbalance between generation and neutralization of the prooxidants. Many well‐known diseases and disorders are related to oxidative stress, such as aging, rheumatoid arthritis, cardiovascular diseases, diabetes, cancer, neurodegenerative diseases (Alzheimer's disease [AD] and Parkinson's disease [PD]), and inflammation. Natural or synthetic antioxidants are capable of inhibiting or delaying the oxidation process using several mechanisms, such as scavenging the free radicals. The antioxidants, therefore, play an important role in preventing or treatment of oxidative stress‐induced disorders (Abeyrathne et al., 2022; Cammisuli et al., 2022; Wang et al., 2016).

The literature review shows that the antioxidant properties of the propolis have been studied and confirmed using DPPH, ORAC, FRAP, ABTS^+^, and β‐carotene /linoleic acid methods (El‐Guendouz et al., 2019; Kocot et al., 2018; Okinczyc et al., 2021). In this research, the antioxidant capacity of the Iranian EEP samples was measured by evaluating their ability to inhibit DPPH‐free radicals. The IC_50_ values corresponding to evaluating the antioxidant activity (Table 2) indicated that the EEP samples with the highest (P9) and lowest (P6) TPC and TFC have the most (IC_50_: 4.62 μg/mL) and least (IC_50_: 1031.57 μg/mL) ability to inhibit DPPH‐free radicals. The antioxidant potency of P8 (IC_50_: 5.02 μg/mL), P4 (IC_50_: 5.64 μg/mL), and P11 (IC_50_: 5.9 μg/mL) samples is almost equal to P9. Considering the IC_50_ value of ascorbic acid (3.89 μg/mL), all the studied EEP samples, except for P6, possess a suitable antioxidant capacity.

The statistical correlation study shows that there is a strong negative correlation between these two parameters (TPC and TFC) and the DPPH results (*R* ^2^ = −.99). The obtained results are consistent with the previous data, which correlate the antioxidant activity of the propolis extracts with the TPC and TFC (da Silva et al., 2006; El‐Guendouz et al., 2018; Socha et al., 2015). There are also published documents demonstrating the positive correlation between the antioxidant capacity of the propolis extracts and their TPC (Ahn et al., 2007; Hamasaka et al., 2004; Kalogeropoulos et al., 2009; Moreira et al., 2008; Wang et al., 2016) or TFC (Isla et al., 2009). There is ample evidence representing the remarkable antioxidant activity of the phenolic compounds especially flavonoids (Abeyrathne et al., 2022; Liu, 2022; Shen et al., 2022).

### 3.2 Anticholinesterase (anti‐AChE and anti‐BuChE) activity

Based on several published papers in recent years, propolis can be considered as a promising therapeutic natural substance to protect the brain and treat neurological injuries and disorders (Ayikobua et al., 2018; Bhargava et al., 2021; Zulhendri, Chandrasekaran, et al., 2021; Zulhendri, Perera, et al., 2021).

The administration of cholinesterase enzymes (AChE and BuChE) inhibitors is the main therapeutic strategy for the symptomatic treatment of mild to moderately severe forms of AD (Colovic et al., 2013; Sharma, 2019). The anticholinesterases are also used to manage other forms of neurological disorders, such as ataxia, PD, dementia with Lewy bodies, senile dementia, vascular dementia, Down's syndrome, Korsakoff disease, and myasthenia gravis (Farmakidis et al., 2018; Kulshreshtha & Piplani, 2016; Mukherjee et al., 2007). Nowadays, there are worldwide research efforts for the design and development of novel cholinesterase inhibitors with more efficacy and fewer side effects (Lotfi et al., 2020; Mariki et al., 2021; Sharma, 2019). The identification of new potent anticholinesterase compounds through the study of natural resources has also attracted a lot of attention (Dey et al., 2017; Dos Santos et al., 2018; Houghton et al., 2006; Mukherjee et al., 2007).

The results corresponding to the evaluation of anti‐AChE and anti‐BuChE activity of the EEP samples are represented in Table 3. As well seen, all the EEP samples are capable of inhibiting cholinesterase enzymes, but their ability to inhibit the enzymes is very different. Among the 12 EEP samples, P4 (IC_50_: 14.37 μg/mL) and P10 (IC_50_: 239 μg/mL) show the highest and lowest potency to inhibit AChE. These two samples with the respective IC_50_ values of 13.05 μg/mL and 155.34 μg/mL also possess the highest and lowest anti‐BuChE activity. The P9 sample is also a potent inhibitor of BuChE and its IC_50_ value for the enzyme (15.06 μg/mL) is very close to that of P4. Whereas, the anti‐AChE activity of P9 (IC_50_: 25.72 μg/mL) is almost 1.8 times less than that of P4. The IC_50_ values of neostigmine for AChE and BuChE inhibition are 0.023 μg/mL and 0.04 μg/mL, respectively. Therefore, the potency of all the propolis extracts to inhibit AChE and BuChE is much less than that of the reference compound.

**TABLE 3:** In vitro inhibition (IC_50_,^a^, μg/mL) of 12 Iranian EEP samples (P1–P12) and the reference compound (neostigmine) on AChE and BuChE.

Sample	IC_50_ for AChE (μg/mL)	IC_50_ for BuCE (μg/mL)	Selectivity for BuChE^b^
P1	45.56 ± 0.020	30.40 ± 0.09	1.5
P2	62.08 ± 0.1	41.91 ± 0.028	1.48
P3	83.79 ± 0.12	66.64 ± 0.034	1.26
P4	14.37 ± 0.028	13.05 ± 0.005	1.1
P5	69.53 ± 0.034	65.52 ± 0.028	1.06
P6	174.06 ± 0.24	128.67 ± 0.18	1.35
P7	154.66 ± 0.13	92.75 ± 0.045	1.67
P8	43.58 ± 0.028	23.69 ± 0.008	1.84
P9	25.72 ± 0.020	15.06 ± 0.004	1.7
P10	239 ± 0.025	155.34 ± 0.17	1.54
P11	44.71 ± 0.028	39.95 ± 0.024	1.12
P12	77.73 ± 0.12	49.98 ± 0.028	1.55
Neostigmine	0.023 ± 0.001	0.04 ± 0.0005	0.57

^a^ Concentration of the extract required for 50% inhibition of AChE or BuChE. The data are shown as mean ± SEM of three experiments.

^b^ AChE IC_50_/BuChE IC_50_.

As shown in Table 3, the anti‐BuChE activity of three EEP samples (P4, P5, and P11) is slightly higher than anti‐AChE activity, but the rest of the samples show a distinct selectivity over BuChE. The data published on the anticholinesterase activity of Algerian propolis methanolic extracts also demonstrated the higher ability of the extracts to inhibit BuChE than AChE (Boulechfar et al., 2019, 2022). Considering that in the late stages of AD, there is a decrease in AChE level and an increase in BuChE level in the brain, selective BuChE inhibitors are of great importance in the treatment of advanced AD (Li et al., 2017).

As shown in Table 6, a strong negative correlation is observed between the TPC and TFC, and the results obtained from the evaluation of the anti‐AChE (*R* ^2^ = −.94) and anti‐BuChE (*R* ^2^ = −.93) of the EEP samples. This means the EEP samples with higher TPC and TFC possess a higher ability to inhibit the cholinesterase enzymes (AChE and BuChE) and vice versa. There are published reports indicating the positive correlation between the anticholinesterase activity and the TPC and TFC of the propolis extracts from Turkey (Baltas et al., 2016) and Morocco (El‐Guendouz et al., 2016). No direct correlation has been observed between anticholinesterase potency and TPC and TFC of the studied Korean EEPs (Wang et al., 2016).

### 3.3 Antibacterial activity

The antibacterial properties of propolis have been well studied and documented (Bouchelaghem, 2022; Przybylek & Karpinski, 2019; Zulhendri, Chandrasekaran, et al., 2021; Zulhendri, Perera, et al., 2021). As explained in the experimental section, the antibacterial activity of the Iranian propolis ethanolic extracts was investigated using two gram‐positive (*B. cereus*, *S. aureus*) and two gram‐negative (*A. baumannii*, *P. aeruginosa*) pathogenic bacteria.

*B. cereus* which exists in soil, different types of raw and processed foods, and vegetables, is an aerobic spore‐forming and a common food poisoning bacterium. This organism generates two types of food poisoning, the emetic and diarrheal syndromes, and an array of systemic and local infections (Schoeni & Wong, 2005; Stenfors Arnesen et al., 2008). *S. aureus* is a facultative anaerobic coccus. Some *S. aureus* strains are capable of producing enterotoxins and causing food poisoning. Symptoms of staphylococcal food poisoning are nausea, vomiting, and abdominal cramps, with or without diarrhea (Argudin et al., 2010; Le Loir et al., 2003). The antibacterial effects of propolis on foodborne bacteria, such as *B. cereus*, *S. aureus*, *Listeria monocytogenes*, *Enterococcus faecalis*, and *Clostridium perfringens* have proposed it as a promising natural preservative (Kim & Chung, 2011; Yang et al., 2017).

*A. baumannii* is an opportunistic bacillus and the major cause of hospital‐acquired infections. *P. aeruginosa* is also an opportunistic rod‐shaped pathogen responsible for nosocomial infections. Due to the rapid emergence of multidrug‐resistant strains of *A. baumannii* and *P. aeruginosa*, the therapeutic strategies for the infections caused by these two pathogens are limited (Kunz Coyne et al., 2022; Nocera et al., 2021). Therefore, the study of natural resources, such as propolis to find and develop new pharmaceutical agents for the treatment of infections caused by *A. baumannii* and *P. aeruginosa* has great importance (Hannan et al., 2015; Meto et al., 2020).

In addition to the determination of MIC (minimum inhibitory concentration) and MBC (minimum bactericidal concentration) (Table 4), the diameter of the growth inhibition halo was calculated for different concentrations of each EEP sample by agar well diffusion method (Table 5).

**TABLE 4:** The minimum inhibitory concentration (MIC) and minimum bactericidal concentration (MBC) of 12 Iranian EEPs for *B. cereus*, *S. aureus*, *A. baumannii*, and *P. aeruginosa*.

Sample	Bacteria							
	*Bacillus cereus*		*Staphylococcus aureus*		*Acinetobacter baumannii*		*Pseudomonas aeruginosa*	
	MIC (μg/mL)	MBC (μg/mL)	MIC (μg/mL)	MBC (μg/mL)	MIC (μg/mL)	MBC (μg/mL)	MIC (μg/mL)	MBC (μg/mL)
P1	52.08 ± 18.04^c^	104.16 ± 36.08^b^	62.5 ± 0.00^bcd^	104.16 ± 36.08^cd^	83.33 ± 36.08^b^	250 ± 0.00^a^	125 ± 0.00^a^	250 ± 0.00^a^
P2	62.5 ± 0.00^bc^	125 ± 0.00^b^	62.5 ± 0.00^bcd^	125 ± 0.00^cd^	83.33 ± 36.08^b^	250 ± 0.00^a^	83.33 ± 36.08^a^	125 ± 0.00^b^
P3	208.33 ± 72.16^a^	208.33 ± 72.16^a^	166.66 ± 72.16^a^	250 ± 0.00^a^	125 ± 0.00^ab^	250 ± 0.00^a^	104.16 ± 36.08^a^	208.33 ± 72.16^ab^
P4	26.04 ± 9.02^c^	104.16 ± 36.08^b^	26.04 ± 9.02^d^	62.5 ± 0.00^d^	62.5 ± 0.00^b^	125 ± 0.00^c^	83.33 ± 36.08^a^	166.66 ± 72.16^ab^
P5	52.08 ± 18.04 ^c^	104.16 ± 36.08^b^	41.66 ± 18.04^cd^	104.16 ± 36.08^cd^	125 ± 0.00^ab^	250 ± 0.00^a^	104.16 ± 36.08^a^	208.33 ± 72.16^ab^
P6	125 ± 0.00^b^	250 ± 0.00^a^	104.16 ± 36.08^b^	208.33 ± 72.16^ab^	166.66 ± 72.16^a^	250 ± 0.00^a^	125 ± 0.00^a^	250 ± 0.00^a^
P7	104.16 ± 36.08^b^	208.33 ± 72.16^a^	83.33 ± 36.08^bc^	166.66 ± 72.16^bc^	125 ± 0.00^ab^	250 ± 0.00^a^	104.16 ± 36.08^a^	208.33 ± 72.16^ab^
P8	26.04 ± 9.02^c^	104.16 ± 36.08^b^	52.08 ± 18.04^bcd^	104.16 ± 36.08^cd^	83.33 ± 36.08^b^	166.66 ± 72.16^bc^	104.16 ± 36.08^a^	166.66 ± 72.16^ab^
P9	31.25 ± 0.00^c^	104.16 ± 36.08^b^	26.04 ± 9.02^d^	62.5 ± 0.00^d^	83.33 ± 36.08^b^	250 ± 0.00^a^	83.33 ± 36.08^a^	166.66 ± 72.16^ab^
P10	125 ± 0.00^b^	125 ± 0.00^b^	104.16 ± 36.08^b^	208.33 ± 72.16^ab^	83.33 ± 36.08^b^	208.33 ± 72.16^ab^	83.33 ± 36.08^a^	166.66 ± 72.16^ab^
P11	52.08 ± 18.04^c^	104.16 ± 36.08^b^	62.5 ± 0.00^bcd^	125 ± 0.00^cd^	83.33 ± 36.08^b^	208.33 ± 72.16^ab^	83.33 ± 36.08^a^	166.66 ± 72.16^ab^
P12	104.16 ± 36.08^b^	208.33 ± 72.16^a^	62.5 ± 0.00^bcd^	125 ± 0.00^cd^	125 ± 0.00^ab^	250 ± 0.00^a^	104.16 ± 36.08^a^	208.33 ± 72.16^ab^

*Note*: abcd: The significant difference of means between rows within column (*p* < .05).

**TABLE 5:** The Diameter of growth inhibition haloes (in millimeters) generated by seven different concentrations of the studied Iranian EEP samples and positive control (ciprofloxacin) measured by agar well diffusion method using four pathogenic bacteria (*B. cereus*, *S. aureus*, *A. baumannii*, and *P. aeruginosa*).

Bacteria Sample		Concentration (μg/mL)						
		500	250	125	62.5	31.25	15.62	7.81
*Bacillus cereus*	Ciprofloxacin	9.00 ± 0.34^a^						
	P1	5.00 ± 0.57^c^	4.33 ± 0.66^d^	3.00 ± 0.21^e^	2.33 ± 0.11^e^	1.66 ± 0.43^f^	1.66 ± 0.23^f^	1.33 ± 0.10^f^
	P2	4.66 ± 0.33^cd^	3.33 ± 0.53^de^	3.00 ± 0.71^e^	2.33 ± 0.24^e^	1.33 ± 0.23^f^	1.00 ± 0.00^f^	0.66 ± 0.09^fg^
	P3	3.66 ± 0.54^d^	3.33 ± 0.25^de^	2.66 ± 0.67^e^	2.00 ± 0.34^ef^	2.00 ± 0.21^ef^	1.33 ± 0.22^f^	1.00 ± 0.31^f^
	P4	6.33 ± 0.56^b^	5.33 ± 0.44^c^	4.66 ± 0.31^cd^	4.33 ± 0.11^d^	3.66 ± 0.76^d^	2.33 ± 0.22^e^	1.66 ± 0.35^f^
	P5	5.33 ± 0.66^c^	4.66 ± 0.32^cd^	4.00 ± 0.00^d^	3.33 ± 0.12^de^	2.33 ± 0.36^e^	1.66 ± 0.45^f^	1.33 ± 0.12^f^
	P6	3.66 ± 0.43^d^	2.66 ± 0.26^e^	2.33 ± 0.15^e^	2.00 ± 0.00^ef^	1.33 ± 0.48^f^	1.00 ± 0.00^f^	0.66 ± 0.37^fg^
	P7	2.66 ± 0.32^e^	2.33 ± 0.44^e^	2.00 ± 0.00^ef^	1.33 ± 0.23^f^	1.00 ± 0.00^f^	1.00 ± 0.00^f^	0.66 ± 0.09^fg^
	P8	7.00 ± 0.65^b^	5.66 ± 0.54^c^	4.00 ± 0.00^d^	3.33 ± 0.16^de^	2.66 ± 0.27^e^	2.33 ± 0.32^e^	1.66 ± 0.44^f^
	P9	7.33 ± 0.55^b^	6.33 ± 0.46^b^	5.66 ± 0.57^c^	4.00 ± 0.00^d^	3.66 ± 0.43^d^	2.66 ± 0.21^e^	2.00 ± 0.00^ef^
	P10	2.66 ± 0.11^e^	2.00 ± 0.00^ef^	1.66 ± 0.44^f^	1.33 ± 0.31^f^	1.00 ± 0.00^f^	0.66 ± 0.21^fg^	0.33 ± 0.11^g^
	P11	6.00 ± 0.35^bc^	5.00 ± 0.00^c^	4.66 ± 0.31^cd^	4.00 ± 0.00^d^	3.00 ± 0.32^e^	2.00 ± 0.00^ef^	1.66 ± 0.11^f^
	P12	2.33 ± 0.35^e^	2.00 ± 0.43^ef^	2.00 ± 0.44^ef^	1.00 ± 0.00^f^	0.66 ± 0.24^fg^	0.66 ± 0.24^fg^	0.33 ± 0.29^g^
*Staphylococcus aureus*	Ciprofloxacin	9.00 ± 0.45^a^						
	P1	4.65 ± 0.57^c^	3.53 ± 0.76^d^	2.66 ± 0.41^e^	1.33 ± 0.21^e^	1.23 ± 0.53^ef^	1.26 ± 0.43^ef^	1.10 ± 0.10^ef^
	P2	3.56 ± 0.53^cd^	2.43 ± 0.45^de^	2.11 ± 0.51^e^	1.33 ± 0.24^e^	1.11 ± 0.23^ef^	0.66 ± 0.00^f^	0.46 ± 0.09^fg^
	P3	2.76 ± 0.54^d^	2.43 ± 0.21^de^	1.75 ± 0.61^e^	1.32 ± 0.32^ef^	1.10 ± 0.21^ef^	0.66 ± 0.22^f^	0.56 ± 0.31^f^
	P4	5.43 ± 0.57^b^	4.53 ± 0.44^c^	3.76 ± 0.51^c^	3.53 ± 0.71^d^	2.56 ± 0.66^d^	1.53 ± 0.42^e^	1.06 ± 0.55^f^
	P5	4.53 ± 0.46^c^	3.76 ± 0.52^c^	3.11 ± 0.40^d^	2.53 ± 0.12^de^	1.73 ± 0.38^e^	0.87 ± 0.65^f^	0.78 ± 0.17^f^
	P6	2.76 ± 0.53^d^	1.76 ± 0.36^e^	1.43 ± 0.25^e^	1.10 ± 0.30^ef^	0.83 ± 0.38^f^	0.83 ± 0.20^f^	0.46 ± 0.37^fg^
	P7	1.76 ± 0.32^e^	1.43 ± 0.44^e^	1.00 ± 0.00^ef^	0.83 ± 0.23^f^	0.74 ± 0.30^fg^	0.74 ± 0.20^f^	0.56 ± 0.09^fg^
	P8	6.00 ± 0.65^b^	4.76 ± 0.58^c^	3.60 ± 0.20^d^	2.53 ± 0.16^de^	1.86 ± 0.27^e^	1.73 ± 0.32^e^	0.86 ± 0.44^f^
	P9	6.53 ± 0.55^b^	5.53 ± 0.36^b^	4.66 ± 0.67^c^	3.27 ± 0.40^d^	2.86 ± 0.43^d^	1.76 ± 0.41^e^	1.32 ± 0.10^ef^
	P10	1.66 ± 0.31^e^	1.65 ± 0.34^ef^	1.00 ± 0.44^ef^	0.83 ± 0.21^f^	1.00 ± 0.00^f^	0.46 ± 0.21^fg^	0.30 ± 0.31^g^
	P11	5.24 ± 0.35^bc^	4.34 ± 0.67^c^	3.46 ± 0.31^d^	3.21 ± 0.50^d^	2.15 ± 0.62^e^	1.20 ± 0.46^ef^	0.56 ± 0.11^fg^
	P12	1.35 ± 0.45^e^	1.21 ± 0.43^ef^	1.21 ± 0.44^ef^	1.24 ± 0.30^ef^	0.46 ± 0.24^fg^	0.56 ± 0.27^fg^	0.23 ± 0.19^g^
*Acinetobacter baumannii*	Ciprofloxacin	11.00 ± 0.55^a^						
	P1	4.00 ± 0.78^e^	3.66 ± 0.56^cd^	3.00 ± 0.58^d^	3.00 ± 0.78^d^	2.33 ± 0.45^d^	1.66 ± 0.62^e^	0.66 ± 0.37^e^
	P2	3.00 ± 0.00^d^	2.66 ± 0.43^d^	2.33 ± 0.35^d^	2.00 ± 0.00^de^	1.33 ± 0.65^e^	1.00 ± 0.00^e^	0.33 ± 0.34^e^
	P3	3.66 ± 0.45^cd^	1.33 ± 0.68^e^	1.00 ± 0.00^e^	1.00 ± 0.67^e^	0.66 ± 0.32^e^	0.33 ± 0.24^e^	0.33 ± 0.41^e^
	P4	4.66 ± 0.32^c^	3.33 ± 0.59^d^	2.66 ± 0.45^d^	2.33 ± 0.32^d^	1.66 ± 0.35^e^	1.33 ± 0.35^e^	1.00 ± 0.00^e^
	P5	3.33 ± 0.67^d^	2.00 ± 0.00^de^	2.00 ± 0.67^de^	1.33 ± 0.56^e^	2.33 ± 0.78^d^	0.66 ± 0.34^e^	0.33 ± 0.27^e^
	P6	1.33 ± 0.32^e^	1.33 ± 0.46^e^	1.66 ± 0.34^e^	1.33 ± 0.65^e^	0.66 ± 0.35^e^	0.66 ± 34^e^	0.33 ± 0.12^e^
	P7	2.33 ± 0.47^d^	1.66 ± 0.77^e^	1.00 ± 0.56^e^	1.33 ± 0.34^e^	0.66 ± 0.45^e^	0.66 ± 0.57^e^	0.33 ± 0.12^e^
	P8	5.33 ± 0.76^b^	5.00 ± 0.65^bc^	3.66 ± 0.53^dc^	3.33 ± 0.42^d^	3.00 ± 0.67^d^	2.00 ± 0.00^de^	1.66 ± 0.32^e^
	P9	5.66 ± 0.56^b^	5.00 ± 0.47^bc^	4.33 ± 0.43^c^	4.00 ± 0.56^c^	3.33 ± 0.36^d^	2.33 ± 0.46^d^	1.66 ± 0.38^e^
	P10	2.66 ± 0.32^d^	2.33 ± 0.68^d^	1.66 ± 0.59^e^	1.33 ± 0.35^e^	1.33 ± 0.24^e^	1.00 ± 0.00^e^	0.33 ± 0.24^e^
	P11	4.66 ± 0.21^c^	3.66 ± 0.46^cd^	4.66 ± 0.53^c^	2.33 ± 0.67^d^	2.00 ± 0.00^de^	1.66 ± 0.43^e^	1.33 ± 0.32^e^
	P12	2.00 ± 0.00^de^	2.00 ± 0.78^de^	1.66 ± 0.36^e^	1.33 ± 0.44^e^	1.00 ± 0.89^e^	0.66 ± 0.43^e^	0.33 ± 0.58^e^
*Pseudomonas aeruginosa*	Ciprofloxacin	10.00 ± 0.61^a^						
	P1	3.55 ± 0.64^c^	3.02 ± 0.46^cd^	2.54 ± 0.26^d^	2.50 ± 0.67^d^	1.84 ± 0.35^d^	1.12 ± 0.43^e^	0.35 ± 0.19^e^
	P2	2.10 ± 0.32^d^	2.14 ± 0.33^d^	1.85 ± 0.54^d^	1.56 ± 0.36^ed^	0.87 ± 0.34^e^	0.58 ± 0.21^e^	0.18 ± 0.14^e^
	P3	3.04 ± 0.36^cd^	0.93 ± 0.54^d^	0.66 ± 0.10^e^	0.66 ± 0.47^e^	0.35 ± 0.22^e^	0.23 ± 0.14^e^	0.23 ± 0.21^e^
	P4	4.26 ± 0.46^c^	2.83 ± 0.47^d^	2.27 ± 0.45^d^	1.93 ± 0.52^d^	1.26 ± 0.35^e^	0.94 ± 0.31^e^	0.75 ± 0.23^e^
	P5	2.75 ± 0.67^d^	1.56 ± 0.67^de^	1.50 ± 0.67^de^	1.00 ± 0.00^e^	1.83 ± 0.68^d^	0.46 ± 0.31^e^	0.23 ± 0.17^e^
	P6	1.00 ± 0.32^e^	0.83 ± 0.46^e^	1.26 ± 0.64^e^	1.00 ± 0.00^e^	0.36 ± 0.27^e^	0.44 ± 0.34^e^	0.18 ± 0.12^e^
	P7	2.00 ± 0.00^d^	1.46 ± 0.77^e^	0.67 ± 0.56^e^	0.93 ± 0.34^e^	0.46 ± 0.35^e^	0.46 ± 0.27^e^	0.13 ± 0.12^e^
	P8	4.83 ± 0.76^b^	4.57 ± 0.65^bc^	3.16 ± 0.33^dc^	2.83 ± 0.42^d^	2.58 ± 0.67^d^	1.67 ± 0.43^de^	1.26 ± 0.32^e^
	P9	5.16 ± 0.59^b^	4.67 ± 0.47^bc^	4.00 ± 0.43^c^	3.67 ± 0.78^c^	2.89 ± 0.36^d^	1.89 ± 0.46^d^	1.26 ± 0.57^e^
	P10	2.26 ± 0.32^d^	1.73 ± 0.78^d^	1.24 ± 0.89^e^	1.00 ± 0.35^e^	0.83 ± 0.54^e^	0.78 ± 0.54^e^	0.13 ± 0.13^e^
	P11	4.26 ± 0.71^c^	3.24 ± 0.46^cd^	4.14 ± 0.53^c^	2.00 ± 0.67^d^	1.45 ± 0.65^de^	1.25 ± 0.33^e^	0.93 ± 0.32^e^
	P12	1.67 ± 0.76^de^	1.57 ± 0.78^de^	1.16 ± 0.76^e^	1.00 ± 0.44^e^	0.78 ± 0.69^e^	0.46 ± 0.43^e^	0.23 ± 0.18^e^

*Note*: abcdefg: the significant difference of means between rows within column (*p* < .05).

In general, different mean MIC values were observed for the studied bacteria. These values are variable between 26.04–208.33 μg/mL for *B. cereus*, 26.04–166.66 μg/mL for *S. aureus*, 62.5–166.66 μg/mL for *A. baumannii*, and 83.33–125 μg/mL for *P. aeruginosa*. Several MIC values below 100 μg/mL including 26.04 μg/mL (P4, P8, and P9), 31.25 μg/mL (P9), 41.66 μg/mL (P5), 52.08 μg/mL (P1, P5, P8, and P11), 62.5 μg/mL (P2, P11, and P12), and 83.33 μg/mL (P7) were observed for *B. cereus* and *S. aureus*. While, only two MIC values below 100 μg/mL including 62.5 μg/mL (P4) and 83.33 μg/mL (P1, P2, P4, and P8–P11) were recorded for *A. baumannii* and *P. aeruginosa*. The mean MBC values range from 104.16–250 μg/mL, 62.5–250 μg/mL, 125–250 μg/mL, and 125–250 μg/mL for *B. cereus*, *S. aureus*, *A. baumannii*, and *P. aeruginosa*, respectively. Two EEPs (P4, P9) show the lowest MBC value of 62.5 μg/mL for *S. aureus*. MBC of several EEPs (P1, P4, P5, and P8, P9, P11) is 104.16 μg/mL for *B. cereus* and *S. aureus*. Only one EEP sample possesses the MBC value of 125 μg/mL for *A. baumannii* (P4), and *P. aeruginosa* (P2). Most of the EEP samples (P1–P3, P5, P5–P7, P9, and P12) exhibit the highest MBC value, 250 μg/mL, for *A. baumannii*, and 166.66 μg/mL (P4, P8–P11) or 208.33 μg/ mL (P3, P5, P7, and P12) for *P. aeruginosa*. According to the obtained results, the antibacterial effects of the propolis ethanolic extracts on the studied gram‐positive bacteria (*B. cereus* and *S. aureus*) are more evident.

As mentioned earlier, the antibacterial activity of the EEP samples was also investigated using the agar well diffusion method. As shown in Table 5, seven different concentrations (7.81–500 μg/mL) of each EEP sample and one concentration (500 μg/mL) of ciprofloxacin (positive control) were studied in this test. An overview of the results indicated that as the concentration of EEP samples increases, the diameter of the growth inhibition halo also increases. The inhibition halo diameter of ciprofloxacin is 9 mm for *B. cereus* and *S. aureus* and 11 and 10 mm for *A. baumannii* and *P. aeruginosa*. The diameters of growth inhibition haloes corresponding to 500 μg/mL concentration of the EEP samples are variable between 2.33–7.33 and 1.35–6.53 mm for *B. cereus* and *S. aureus*, respectively. These values range from 1.33 to 5.66 mm and from 1 to 5.16 mm for *A. baumannii* and *P. aeruginosa*. For both gram‐positive bacteria, P9 and P12 and for both gram‐negative bacteria P9 and P6 show the most and least ability to inhibit bacterial growth, correspondingly. The diameters of inhibition haloes created by the second active sample (P8) are 7 mm (*B. cereus*), 6 mm (*S. aureus*), 5.33 mm (*A. baumannii*), and 4.83 mm (*P. aeruginosa*). According to the results, the examined Iranian EEPs exhibit greater antibacterial activity against the gram‐positive bacteria (*B. cereus* and *S. aureus*). There is abundant evidence manifesting that the antibacterial properties of propolis influence gram‐positive bacteria more than gram‐negative bacteria (Almuhayawi, 2020; Przybylek & Karpinski, 2019; Silva‐Carvalho et al., 2015).

The existence of a strong positive correlation between TPC and TFC of the EEPs and the results obtained from agar well diffusion 1 (*R* ^2^ = .81), 2 (*R* ^2^ = .83), 3 (*R* ^2^ = .85), and 4 (*R* ^2^ = .82) (Table 6) demonstrates that the EEP samples with higher TPC and TFC inhibit the growth of pathogenic bacteria with more power. These results are consistent with some previously published reports (da Silva et al., 2006; Górniak et al., 2019; Inui et al., 2014; Yuan et al., 2021).

**TABLE 6:** The Spearman correlations between studied parameters calculated by SPSS software.

	TPC	TFC	DPPH	Anti‐AChE	Anti‐BuChE	Agar well diffusion 1^a^	Agar well diffusion 2^b^	Agar well diffusion 3^c^	Agar well diffusion 4^d^
TPC	1.00	1.00**	−0.99**	−0.94**	−0.93**	0.81**	0.83**	0.85**	0.82**
TFC	1.00**	1.00	−0.99**	−0.94**	−0.93**	0.81**	0.83**	0.85**	0.82**
Anti‐AChE	−0.94**	−0.94**	0.96**	1.00	0.99**	−0.87**	−0.89**	−0.86**	−0.82**
Anti‐BuChE	−0.93**	−0.93**	0.96**	0.99**	1.00	−0.81**	−0.82**	−0.84**	−0.78**
DPPH	−0.99**	−0.99**	1.00	0.96**	0.96**	−0.81**	−0.84**	−0.85**	−0.82**
Agar well diffusion 1	0.81**	0.81**	−0.81**	−0.87**	−0.81**	1.00	0.99**	0.90**	0.87**
Agar well diffusion 2	0.83**	0.83**	−0.84**	−0.89**	−0.82**	0.99**	1.00	0.91**	0.88**
Agar well diffusion 3	0.85**	0.85**	−0.85**	−0.86**	−0.84**	0.90**	0.91**	1.00	0.99**
Agar well diffusion 4	0.82**	0.82**	−0.82**	−0.82**	−0.78**	0.87**	0.88**	0.99**	1.00

^a^ *Bacillus cereus*.

^b^ *Staphylococcus aureus*.

^c^ *Acinetobacter baumannii*.

^d^ *Pseudomonas aeruginosa*.

**Correlation is significant at the 0.01 level (two‐tailed).

### 3.4 Molecular docking

**FIGURE 1:**
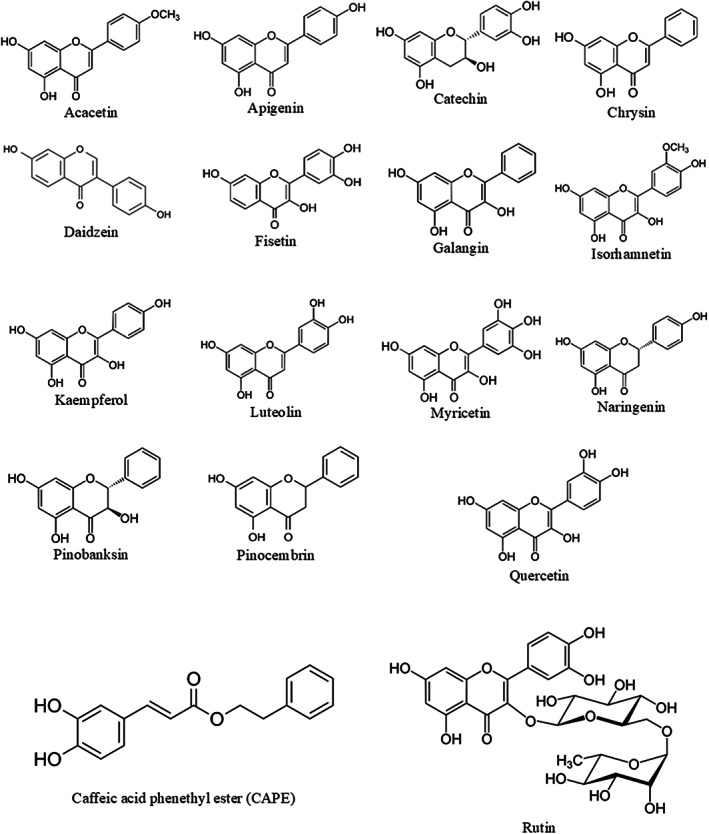
The structure of 17 well‐known propolis flavonoids.

As implied before, the propolis flavonoid composition, which is responsible for many of its biological and medicinal properties, is used as a criterion for the assessment of propolis quality (Huang et al., 2014; Wang et al., 2016). Scientific reports indicate the anticholinesterase activity of the flavonoid compounds (Dzoyem et al., 2017; Khan et al., 2009, 2018). Considering that the results obtained from the current research also represent a strong positive correlation between the anticholinesterase activity of EEP samples and their TFC, it was decided to investigate the interaction of 17 well‐known propolis flavonoids (Kocot et al., 2018; Pasupuleti et al., 2017; Zhang et al., 2021) with the active site gorge of the cholinesterase enzymes (AChE and BuChE) by molecular docking studies. The structure of these flavonoid compounds is illustrated in Figure 1.

The AChE docking results demonstrated that all 17 flavonoid compounds have the ability to interact with the active site gorge of the enzyme and their best binding energy varies between −8.9 and −7 kcal/mol. The lowest docking energy is corresponding to rutin and the highest is related to fisetin (Table 7). Table 8 exhibits the amino acid residues of AChE that play a role in the binding of each flavonoid to the enzyme by creating hydrogen or hydrophobic interactions.

**TABLE 7:** The best docking energies obtained from the study of the interaction of 17 propolis flavonoids with cholinesterase enzymes.

Flavonoid compound		The best docking energy for AChE (kcal/mol)	The best docking energy for BuChE (kcal/mol)
1	Acacetin	−8	−8.3
2	Apigenin	−7.8	−8.8
3	CAPE	−8.2	−8.2
4	Catechin	−7.7	−8.5
5	Chrysin	−8.1	−8.6
6	Daidzein	−7.5	−8.3
7	Fisetin	−7	−8.3
8	Galangin	−7.4	−8.6
9	Isorhamnetin	−7.3	−8.1
10	Kaempferol	−7.3	−8.5
11	Luteolin	−7.9	−9
12	Myricetin	−7.3	−8.6
13	Naringenin	−7.9	−8.7
14	Pinobanksin	−8.2	−8.6
15	Pinocembrin	−8.1	−8.6
16	Quercetin	−7.4	−8.4
17	Rutin	−8.9	−10.8

**TABLE 8:** The amino acid residues involved in the interaction of propolis flavonoid compounds with AChE identified with LigPlot software.

Compound		Amino acid residues involved in the AChE‐flavonoid interaction	
		Hydrogen bonds	Hydrophobic interactions
1	Acacetin	Trp286	Tyr72, Tyr124, His287, Leu289, Phe297, Tyr341, Phe338
2	Apigenin	Tyr72, Tyr124	Trp286, Ser293, Phe295, Phe297, Phe338, Tyr341
3	CAPE	Tyr124, Tyr133, Glu202	Asp74, Trp86, Gly120, Gly121, Gly126, Trp286, Phe295, Phe297, Tyr337, Phe338, Tyr341
4	Catechin	Tyr72, Tyr124, Trp286, Phe295	His287, Ser293, Val294, Phe297, Phe338, Tyr341
5	Chrysin	Tyr72, Trp286	Tyr124, Leu289, Ser293, Phe295, Phe297, Phe338, Tyr341
6	Daidzein	Tyr124, Trp286	Tyr124, Leu289, Ser293, Phe295, Phe297, Phe338, Tyr341
7	Fisetin	Tyr72, Asp74, Tyr124	Thr75, Leu76, Trp286, Ser293, Phe295, Phe297, Tyr341
8	Galangin	Tyr124	Trp286, Glu292, Ser293, Val294, Phe295, Phe297, Phe338, Tyr341
9	Isorhamnetin	Tyr124	Trp286, His287, Leu289, Glu292, Ser293, Val294, Phe295, Phe297, Phe338, Tyr341
10	Kaempferol	Tyr124	Trp286, Glu292, Ser293, Val294, Phe295, Phe297, Phe338, Tyr341
11	Luteolin	Tyr72, Tyr124, Trp286, Phe295	Leu289, Val294, Phe297, Phe338, Tyr341
12	Myricetin	Asn87, Glu202, Ser203, Ser125, Tyr337, His447	Asp74, Thr83, Trp86, Gly120, Gly121, Gly122, Tyr124, Phe295, Phe297, Phe338
13	Naringenin	Tyr72, Tyr124	Trp286, Leu289, Ser293, Phe295, Phe297, Phe338, Tyr341
14	Pinobanksin	Tyr72, Ser293, Tyr341	Tyr124, Trp286, Phe295, Phe297
15	Pinocembrin	Tyr72	Tyr124, Trp286, Leu289, Ser293, Phe295, Phe297, Phe338, Tyr341
16	Quercetin	Tp286, Ser293, Phe295, Arg296, Tyr341	Hi287, Val294, Phe297, Phe338
17	Rutin	Asp74, Thr75, Tyr124, His287, Gln291	Tyr72, Leu76, Trp286, Leu289, Glu292, Ser293, Phe295, Phe297, Phe338, Tyr341

The AChE active site gorge consists of several parts: catalytic triad (Ser203, Glu334, and His447), acyl‐binding pocket (Trp236, Phe295, Phe297, and Phe338), choline‐binding pocket (Trp86, Glu202, and Tyr337), peripheral anionic site (PAS) (Tyr72, Asp74, Tyr124, Trp286, and Tyr341), and oxyanion hole (Gly121, Gly122, and Ala204) (Atanasova et al., 2015; Damuka et al., 2020; Kua et al., 2003; Wiesner et al., 2007; Zhang et al., 2002).

**FIGURE 2:**
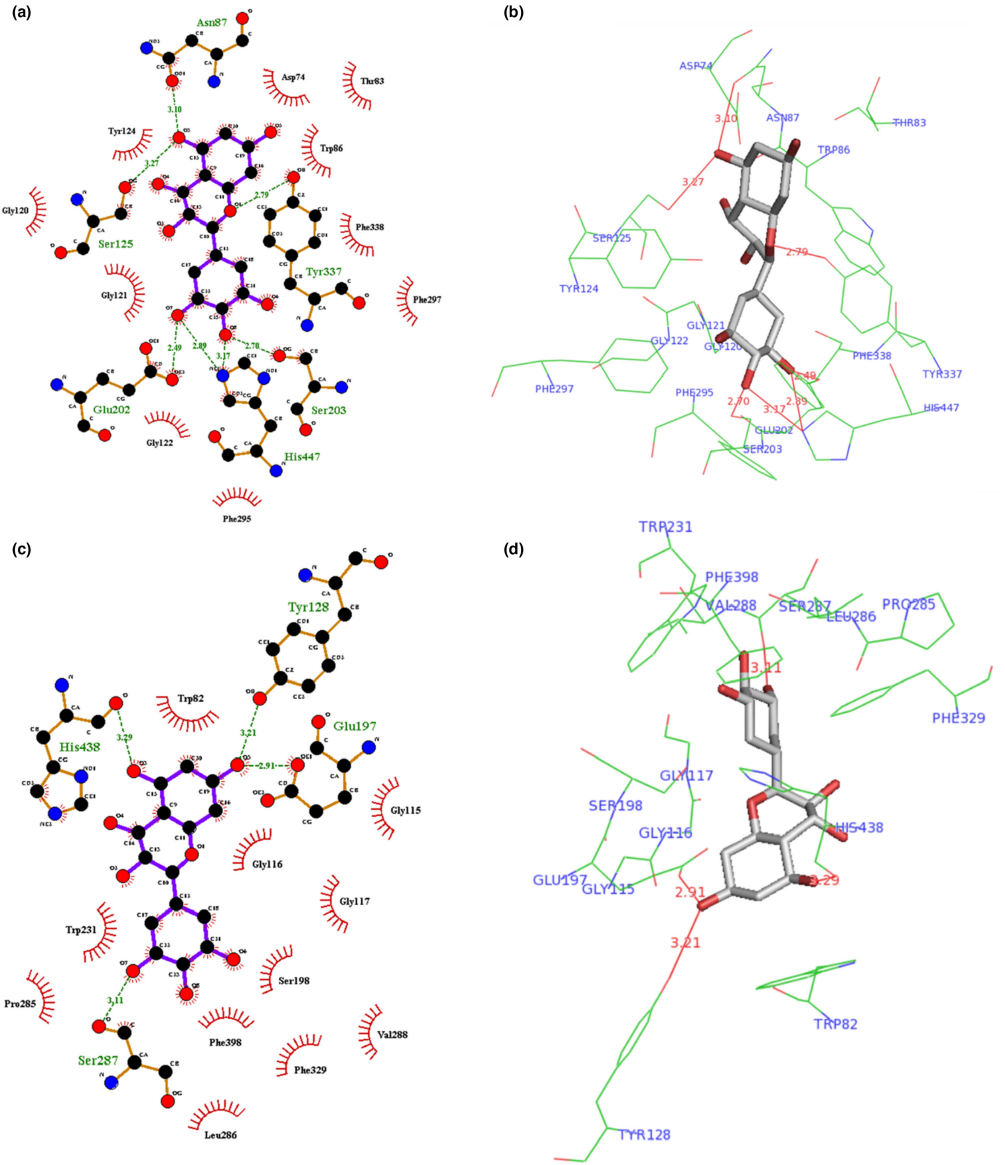
Binding mode of the best docking conformation of myricetin to the active site gorge of AChE (a, b) and BuChE (c, d) prepared using LigPlot and PyMol softwares. The hydrogen bonds are illustrated by dashed green and solid red lines in LigPlot and PyMol, respectively. The amino acid residues participating in the hydrophobic interactions are represented by red half‐moons in the LigPlot images.

As it is clear from the results, several amino acids involved in the binding process are similar. All the compounds interact with PAS and acyl‐binding pocket. The PAS is an allosteric site located near the entrance of the active site gorge. The acyl pocket that is localized near the depth of the gorge is involved in binding to the acetyl group of the substrate (acetylcholine). Among the 17 flavonoids, only caffeic acid phenethyl ester (CAPE) and myricetin interact with the deeper parts of the active site gorge. These two compounds interact with the choline‐binding pocket (responsible for binding to the choline group of acetylcholine) and the oxy‐anion hole, which are located in the deeper areas of the gorge compared to the acyl packet. Myricetin also interacts with two amino acid residues from the catalytic triad (located at the bottom of the gorge) and therefore its interaction with the enzyme is unique compared to other studied flavonoid compounds. For example, the interaction mode of the best docking conformation of myricetin with the AChE active site gorge is shown in Figure 2a,b.

The docking results corresponding to the interaction of flavonoids with BuChE indicated that all the studied compounds interact with the BuChE active site gorge with considerable binding energy (Table 6). Among the 17 studied flavonoids, rutin and isorhamnetin bind to BuChE with the highest (−10.8 kcal/mol) and lowest (−8.1 kcal/mol) affinity, respectively.

The active site gorge of BuChE is also composed of the catalytic triad (Ser198, Glu325, and His438), acyl‐binding pocket (Trp231, Leu286, and Val288), choline‐binding pocket (Trp82 and, Glu197), PAS (Asp70, Phe329, and Tyr332), and oxyanion hole (Gly116 and Gly117) (Brus et al., 2014; Macdonald et al., 2012).

The amino acid residues participating in the binding of BuChE to the flavonoids are represented in Table 9. As well seen, all the studied flavonoid compounds interact with PAS and choline‐binding pocket. Most of the studied propolis flavonoids also interact with one or two amino acid residues from the catalytic triad (except for daidzein) and oxyanion hole (except for daidzein, luteolin, naringenin, and quercetin). The acyl‐binding pocket is also involved in the interaction of catechin, chrysin, fisetin, galangin, isorhamnetin, kaempferol, myricetin, pinobanksin, and pinocembrin with the BuChE gorge area. For instance, the binding mode of the best docking pose of myricetin with the BuChE active site gorge is represented in Figure 2c,d.

**TABLE 9:** The amino acid residues contributing in the interaction of propolis flavonoid compounds with BuChE determined using LigPlot software.

Compound		Amino acid residues involved in the BuChE‐flavonoid interaction	
		Hydrogen bonds	Hydrophobic interactions
1	Acacetin	Gly115, Thr120, Glu197	Trp82, Gly116, Gly121, Tyr128, Pro285, Ala328, Phe329, Tyr332, His438, Gly439
2	Apigenin	Trp82, Thr120, Tyr128, Ala328	Gly115, Gly116, Glu197, Tyr332, Trp430, Met437, His438
3	CAPE	Trp82, Gly115, Tyr128, Tyr440	Asp70, Gly116, Glu197, Ala328, Phe329, Tyr332, Tyr430, His438
4	Catechin	Gly116, Gly117, Ser198, Leu286, Ser287	Trp82, Gly115, Trp231, Val288, Phe329, Phe398, His438
5	Chrysin	Glu197	Trp82, Gly116, Gly117, Ser198, Trp231, Leu286, Phe329, Phe398, His438
6	Daidzein	Tyr128, Glu197	Trp82, Gly115, Ala328, Phe329, Tyr332
7	Fisetin	Gly116, Gly117, Ser198, Leu286, Leu287	Trp82, Trp231, Val288, Phe329, Phe398, His438
8	Galangin	Gly116, Gly117, Ser198	Trp82, Gly115, Trp231, Leu286, Phe329, Phe398, His438
9	Isorhamnetin	Glu197, Leu286	Trp82, Gly115, Gly116, Gly117, Ser198, Trp231, Ser287, Phe329, Phe398, His438
10	Kaempferol	Gly116, Gly117, Ser198, Leu286	Trp82, Gly115, Trp231, Val288, Phe329, Phe398, His438
11	Luteolin	Asn68, Asp70, Trp82, Asn83, Tyr128, Glu197, His438	Ile69, Gly115, Thr120, Gly439
12	Myricetin	Tyr128, Glu197, Ser287, His438	Trp82, Gly115, Gly116, Gly117, Ser198, Trp231, Pro285, Leu286, Val288, Phe329, Phe398
13	Naringenin	Asn68, Tyr128, Glu197, His438	Ile69, Asp70, Trp82, Asn83, Gly115, Thr120, Gly439
14	Pinobanksin	Gly116, Gly117, Ser198	Trp82, Gly115, Trp231, Leu286, Phe329, Tyr332, Phe398, His438
15	Pinocembrin	Glu197	Trp82, Gly116, Gly117, Ser198, Trp231, Leu286, Phe329, Phe398, His438
16	Quercetin	Asn68, Asp70, Asp83, Glu197, His438	Ile69, Trp82, Thr120, Tyr332
17	Rutin	Asn68, Asp70, Trp82, Asp83, Gly115, Tyr128, Glu197, Ser198, Pro285	Ile69, Gly116, Gly117, Thr120, Ala328, Phe329, Trp430, Ile442, His438

According to the docking results, all the studied flavonoids bind to the gorge region of both enzymes with high affinity (Table 7). Analysis of the amino acid residues participating in the interaction process (Tables 8 and 9) shows that the allosteric site (PAS) and at least one of the active site parts (the catalytic triad and the substrate‐binding sites including choline and acyl binding pockets) are involved in the binding of the propolis flavonoids to the active site gorge of the cholinesterase enzymes. Based on previously published papers, the generation of the β‐amyloid (Aβ) fibrils in AD brains accelerates through a noncatalytic manner of the AChE PAS region (Bartolini et al., 2003). Therefore, the inhibitors that can bind concurrently to both active site and PAS (dual‐site inhibitors of AChE), besides increasing acetylcholine levels in AD brains, can decelerate the process of Aβ aggregation. Therefore, the AChE dual‐site inhibitors have attracted a lot of attention for more efficient treatment of AD (Zueva et al., 2019).

It is noteworthy, there is ample evidence demonstrating the neuroprotective effects of all the flavonoids studied in this research (Balaha et al., 2021; Bastianetto et al., 2006; Budzynska et al., 2019; Chen et al., 2017; Dourado et al., 2020; Hamdi et al., 2021; Hussein et al., 2018; Jamali‐Raeufy et al., 2019; Kempuraj et al., 2021; Khan et al., 2019; Kim et al., 2012; Liu et al., 2015; Nabavi et al., 2015, 2016; Nouri et al., 2019; Tao et al., 2018; Zheng et al., 2021).

## 4 CONCLUSION

This is the first comprehensive research on the biological properties of the EEP samples collected from several different regions of Iran. The results showed that half of the examined Iranian EEPs are a rich source of phenolic and flavonoid compounds (P9, P8, P11, P4, P1, and P2) and all the 12 studied EEPs, except for P6, exhibit suitable antioxidant activity. The results obtained from the evaluation of the anticholinesterase activity indicated that all the EEP samples are capable of inhibiting the cholinesterase enzymes (AChE and BuChE), but most of them show a distinct selectivity over BuChE. The antibacterial activity of the Iranian EEPs was investigated using four pathogenic bacteria (*B. cereus*, *S. aureus*, *A. baumannii*, and *P. aeruginosa*). The results demonstrated that the antibacterial properties of propolis are more effective on the studied gram‐positive bacteria (*B. cereus*, *S. aureus*). The correlation results indicated that there is a strong positive correlation between the TPC and TFC of the EEP samples and their antioxidant, anticholinesterase, and antibacterial properties. Considering that there are abundant reports on the anticholinesterase activity of flavonoids and the obtained results demonstrate a strong positive correlation between the anticholinesterase activity of EEP samples and their TFC, the interaction of 17 propolis flavonoids with the cholinesterase enzymes (AChE and BuChE) was studied using molecular docking. The docking results showed that all the flavonoids bind to the active site gorge of the enzymes with high affinity. Summing up, the results suggest that Iranian propolis is a rich source of phenolic and flavonoid compounds and has excellent potential for further studies. As a future perspective, to provide information about the relationship between composition and the studied biological properties of the Iranian EPPs, GC–MS analysis of the most active extracts could be performed. The compounds responsible for the biological effects of the Iranian propolis extracts could be also identified, isolated, and purified using the laboratory methods, such as high‐performance liquid chromatography (HPLC).

## AUTHOR CONTRIBUTIONS

**Shahnaz Fathi Hafshejani:** Formal analysis (supporting); investigation (lead); methodology (supporting); project administration (supporting); resources (supporting); validation (supporting); visualization (supporting); writing – original draft (supporting); writing – review and editing (supporting). **Elham Rezvannejad:** Conceptualization (lead); formal analysis (supporting); investigation (supporting); methodology (supporting); project administration (supporting); resources (supporting); supervision (lead); validation (supporting); visualization (supporting); writing – original draft (supporting); writing – review and editing (supporting). **Mojtaba Mortazavi:** Formal analysis (supporting); resources (supporting); validation (supporting); writing – original draft (supporting); writing – review and editing (supporting). **Ali Riahi‐Madvar:** Resources (equal); validation (supporting); visualization (supporting); writing – original draft (equal); writing – review and editing (equal). **Safa Lotfi:** Conceptualization (lead); formal analysis (supporting); funding acquisition (lead); investigation (supporting); methodology (lead); project administration (lead); resources (supporting); supervision (lead); validation (lead); visualization (supporting); writing – original draft (lead); writing – review and editing (lead).

## CONFLICT OF INTEREST STATEMENT

The authors declare no conflict of interest.

## Data Availability

Data are available on request from the authors.
